# Developing and Evaluating Poultry Preening Behavior Detectors via Mask Region-Based Convolutional Neural Network

**DOI:** 10.3390/ani10101762

**Published:** 2020-09-28

**Authors:** Guoming Li, Xue Hui, Fei Lin, Yang Zhao

**Affiliations:** 1 Department of Agricultural and Biological Engineering, Mississippi State University, Starkville, MS 39762, USA; gl565@msstate.edu; 2 College of Energy and Intelligent Engineering, Henan University of Animal Husbandry and Economy, Zhengzhou 450011, China; xh138@msstate.edu; 3 Department of Electrical and Computer Engineering, Mississippi State University, Starkville, MS 39762, USA; mojiamenke123@gmail.com; 4 Department of Animal Science, The University of Tennessee, Knoxville, TN 37996, USA

**Keywords:** poultry, cage-free, preening behavior, mask R-CNN, residual network

## Abstract

**Simple Summary:**

Preening is poultry grooming and comfort behavior to keep plumages in good conditions. Automated tools to continuously monitor poultry preening behaviors remain to be developed. We developed and evaluated hen preening behavior detectors using a mask region-based convolutional neural network (mask R-CNN). Thirty Hy-line brown hens kept in an experimental pen were used for the detector development. Different backbone architectures and hyperparameters (e.g., pre-trained weights, image resizers, etc.) were evaluated to determine the optimal ones for detecting hen preening behaviors. A total of 1700 images containing 12,014 preening hens were used for model training, validation and testing. Our results show that the final performance of detecting hen preening was over 80% for precision, recall, specificity, accuracy, F1 score and average precision, indicating decent detection performance. The mean intersection over union (MIOU) was 83.6–88.7%, which shows great potential for segmenting objects of concern. The detectors with different architectures and hyperparameters performed differently for detecting preening birds and thus we need to carefully adjust these parameters to obtain a robust deep learning detector. In summary, deep learning techniques may have a great ability to automatically monitor poultry behaviors and assist welfare-oriented poultry management.

**Abstract:**

There is a lack of precision tools for automated poultry preening monitoring. The objective of this study was to develop poultry preening behavior detectors using mask R-CNN. Thirty 38-week brown hens were kept in an experimental pen. A surveillance system was installed above the pen to record images for developing the behavior detectors. The results show that the mask R-CNN had 87.2 ± 1.0% MIOU, 85.1 ± 2.8% precision, 88.1 ± 3.1% recall, 95.8 ± 1.0% specificity, 94.2 ± 0.6% accuracy, 86.5 ± 1.3% F1 score, 84.3 ± 2.8% average precision and 380.1 ± 13.6 ms·image^−1^ processing speed. The six ResNets (ResNet18-ResNet1000) had disadvantages and advantages in different aspects of detection performance. Training parts of the complex network and transferring some pre-trained weights from the detectors pre-trained in other datasets can save training time but did not compromise detection performance and various datasets can result in different transfer learning efficiencies. Resizing and padding input images to different sizes did not affect detection performance of the detectors. The detectors performed similarly within 100–500 region proposals. Temporal and spatial preening behaviors of individual hens were characterized using the trained detector. In sum, the mask R-CNN preening behavior detector could be a useful tool to automatically identify preening behaviors of individual hens in group settings.

## 1. Introduction

The public and industry have expressed increasing concerns about poultry welfare [1,2]. The performance of natural behaviors is commonly used as a criterion in determination of poultry welfare [3]. Preening is one of natural behaviors of poultry and important for keeping plumages well-groomed in both natural and artificial conditions [4]. During preening, birds use their beaks to distribute lipid-rich oil from the uropygial glands to their feathers, while simultaneously removing and consuming parasites [5,6]. Preening, as a preventive body-surface maintenance behavior, could take a large time budget (~13%) out of the total behavior repertoire of Red Jungle fowl [7], thus being unignorable for welfare evaluation. Proper preening behavior responses help to interpret bird status responding to surroundings. Overall time spent preening and number of preening bouts could reflect environment appropriateness for birds. For example, preening is performed whenever there is nothing more important to do and birds in cages showed more time spent preening than those in nature (26% vs. 15%) [5]. Rearing birds in cages may increase bird boredom and not be suitable for bird welfare. The duration and frequency of preening of individual birds could imply their pleasure/frustration status. Birds having no access to resources (e.g., feeder) may feel frustrated and typically perform short-term and frequent preening [8]. The number of simultaneously preening birds could be an indicator of space sufficiency. If allocated space is not enough for all birds to preen simultaneously, high-ranking birds in social groups have priority to preen first and subordinate ones may need to wait [9]. Spatial distribution of preening birds could help to judge sufficiency of resource allowance as well. For instance, if birds could not access feeders due to insufficient feeder allowance, they would preen near the feeders to displace the mild frustration [4]. These are valuable responses for welfare-oriented poultry production and manually collecting these responses could be time- and labor-consuming. However, there is no available automated tool to extract these preening behavior responses. Precision poultry farming techniques may provide availability to automatically obtain these responses, as various sensors and computer tools have been utilized to detect poultry behaviors [10,11]. Convolutional neural network (CNN) is another potential technology for poultry behavior detection.

Convolutional neural networks have been widely utilized for object detection in agricultural applications [12,13]. With sufficient training, the CNN detectors could precisely detect objects of concern in various environments [14]. Meanwhile, the CNN detectors can be integrated into various vision systems to detect objects non-invasively, which is suitable to detect natural behaviors of poultry without extra interferences. The detection performance of the CNNs is various with architectures. Among them, the mask region-based CNN (mask R-CNN) is an extensive network of faster R-CNN [15]. It was used for detecting pig mounting behaviors [16], apple flowers [13], strawberries [17] and so forth and obtained robust performance on those applications. Besides mask R-CNN, our team also applied single shot detector (SSD), faster R-CNN and region-based fully convolutional network (R-FCN) for detecting floor eggs in cage-free hen housing systems [14]. But from the previous paper [15] and our preliminary test, the mask R-CNN outperformed these network architectures with regard to accuracy because it retained as much object information as possible. Hence, it was selected to detect hen preening behaviors in this case.

Mask R-CNN contains a great number of hyperparameters for training and appropriately tuning/modifying the model is important to develop a robust detector in a customized dataset. The residual network (ResNet) is proposed by He et al. [15] and used as a backbone for the mask R-CNN. Various designs and depths of the ResNets can influence speed and quality for extracting features of input images. Some commonly-used CNN models contain considerable weights that were trained with some benchmark datasets, such as common objects in context ‘COCO’ [18] and ImageNet [19]. The weights pre-trained with COCO and ImageNet dataset were hereafter named as pre-trained COCO weights and pre-trained ImageNet weights. To apply the models into customized datasets, one efficient solution is to transfer the pre-trained weights learned previously into parts of the model and only trained the rest parts. Such transfer learning could save training time and simultaneously not compromise network performance [20]. Before developing deep learning models, image resizers are typically used to uniform sizes of input images in benchmark datasets, in which sizes of images are various due to different photographing conditions. Inappropriate resizing strategies may downgrade detection performance. For example, resizing large images into small ones may increase processing speed but risk missing small objects in the resized images [21]; and enlarging small images into large ones with changed length-to-width ratios could distorted shapes and features of objects of concern [22]. Insufficient region proposals may lead to missing target objects while excessive proposals may downgrade processing speed [23]. However, it is uncertain which backbone architecture is better for detecting preening birds and which hyperparameters are more efficient to develop the detectors.

The objective of this research was to develop mask R-CNN preening behavior detectors using brown hens as examples. The brown hens lay brown-shell eggs accounting for a large share (>90% in Europe and >70% in China) of the global egg market [24]. The backbone architecture and hyperparameters, including pre-trained weight, image resizer and regions of interest (ROI), were modified to construct an optimal detector for the detection purpose. The backbone architectures of residual networks (ResNet) were ResNet18, ResNet34, ResNet50, ResNet101, ResNet152 and ResNet1000. The trainings included without pre-trained weights, with the pre-trained COCO weights and with the pre-trained ImageNet weights. The modes of image resizers were ‘None,’ ‘Square’ and ‘Pad64′. Numbers of ROIs were 30, 100, 200, 300, 400 and 500. With the trained detector, hen preening behaviors were quantified as well.

## 2. Materials and Methods

### 2.1. Housing, Animals and Management

The experiment was conducted at the U.S. Department of Agriculture (USDA) Poultry Research Unit at Mississippi State, USA and all procedures in this experiment were approved by the USDA-ARS Institutional Animal Care and Use Committee at Mississippi State, USA. Thirty Hy-line Brown hens at 38 weeks of age were placed in a pen, measuring 2.5 m long × 2.2 m wide. Nest boxes, feeders and drinkers were equipped in the pen. Fresh litter was spread on the floor before bird arrival. Commercial feed was provided ad libitum. Temperature, light program and light intensity were, respectively, set to 24 ℃, 16L:8D (light ON at 6:00 am and OFF at 10:00 pm) and 20 lux at bird head level.

### 2.2. Data Acquisition

A night-vision network camera (PRO-1080MSB, Swann Communications U.S.A Inc., Santa Fe Springs, LA, USA) was mounted in the middle of the pen and at ~2 m above the ground to capture top-view videos. Hen activity was continuously monitored and videos were stored in a digital video recorder (DVR-4580, Swann Communications U.S.A Inc., Santa Fe Springs, LA, USA). The video files were recorded with a resolution of 1280 × 720 pixels at a sample rate of 25 frames per second (fps) and converted to image files (.jpg) using Free Video to JPG Converter (ver. 5.0).

### 2.3. Preening Behavior Definition and Labelling

**Figure 1:**
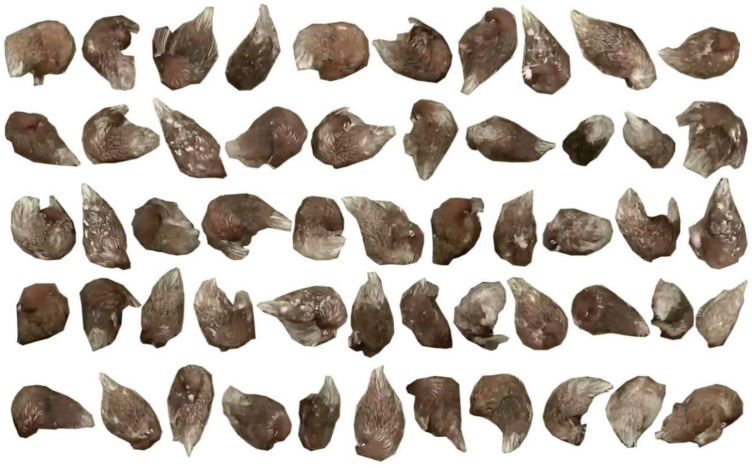
Sample pictures of preening hens. The preening birds were manually cropped from original images.

The definition of preening was that a bird grooms its feathers on different body parts, including breast, throat, belly, shoulder, wing, back, tail and vent [6,25,26,27]. Based on the definition, we manually labeled each preening hen that had the features in Figure 1. It should be noted that this study examined preening behavior with beak only and the preening behavior with foot [26] was not considered. A total of 48 h of videos, 16 h in one day, was used. Images with at least 1-min intervals were selected [12] and the images containing preening hens were used for the labelling, resulting in totally 1700 images from three-day videos. The labelling was conducted in an open-source labeling software (VGG Image Annotator, VIA 2.0.4). A protocol of labeling preening birds that had the features in the preening definition was set. The dataset was split into two parts and two experienced labelers labeled respective parts of images following the protocol. Then they mutually checked the labeled results to ensure that the labels were correct.

### 2.4. Network Description

**Figure 2:**
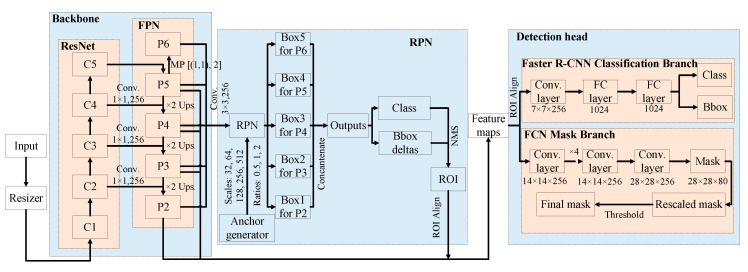
Network structure of mask region-based convolutional neural network (mask R-CNN). ResNet is a residual network; FPN is a feature pyramid network; RPN is a region proposal network; ROI is region of interest; NMS is non-maximum suppression; FC layer is fully-connected layer; Bbox is bounding box; FCN is fully-connected network; C1–C5 are convolutional stages 1 to 5 in the ResNet; P2–P6 are feature maps in the FPN; Box1–Box5 are proposed boxes with various scales and ratios after the RPN; Conv. 1 × 1,256 is the convolution with the kernel size of (1, 1) and depth of 256; MP [(1, 1), 2] is max pooling with the size of (1, 1) and stride of 2; ×2 Ups. is upsampling with the size of (2, 2); Conv. 3 × 3 × 256 is the convolution with the kernel size of (3, 3) and depth of 256; 7 × 7 × 256 is the size (length of 7, width of 7 and depth of 256) of convolution layers; 1024 is the number of neurons in the FC layer; 14 × 14 × 256 is the size (length of 14, width of 14 and depth of 256) of convolution layers; ×4 is the repeated operations of the previous layer for 4 times; 28 × 28 × 256 is the size (length of 28, width of 28 and depth of 256) of convolution layers; 28 × 28 × 80 are 80 target masks with the size of 28 in length and 28 in width.

The mask R-CNN consists of a backbone to extract features from an input image, a region proposal network (RPN) to propose ROI and a detection head for object detection and instance segmentation (Figure 2).

Each input image is first resized into a proper size using an image resizer. A ResNet and a feature pyramid network (FPN) are used to construct the backbone to extract features from the resized image. The ResNet is a bottom-up convolution network and divided into five stages of convolutions (C1–C5) [15]. With higher stages of convolution, the sizes of resultant maps become smaller and higher-level semantics are retained. The FPN is a top-down convolution network and generates five scales of feature maps (P2–P6), which are resulted from the C2–C5 maps, respectively. The C2–C5 and P2–P6 maps are laterally connected with a convolution of 1 × 1 × 256 and up-sampling with the size of (2, 2). The P6 map is processed from the P5 map with a max pooling of [(1, 1), 2]. The ResNet-FPN structure facilitates the extraction of both lower- and higher-level semantics, which are critical for instance segmentation with regards to objects having various scales in an image. The P2–P5 maps are concatenated to form feature maps for detection head, while the P2–P6 maps are combined and go through a convolution of 3 × 3 × 256 to process a map for RPN.

In the RPN, an anchor generator generates the anchors with 5 scales of 32, 64, 128, 256 and 512 and 3 ratios of 0.5, 1 and 2. These anchors are tiled onto the map generated from the P2–P6 maps and then a series of candidate boxes synthesized with objectness and bounding box deltas are proposed. With the non-maximum suppression (NMS) rule, unnecessary boxes are filtered out and ROIs are retained. The ROIs are finally projected onto the feature maps to position objects of interest using ROI Align operation. The ROI Align uses the bilinear function to maintain float coordinates and makes pixel-wise prediction more accurate than the ROI Pooling in the faster R-CNN, in which float coordinates are typically quantized and valuable pixel information may lose.

The detection head comprises three branches that are object classification branch, bounding box regression branch and object instance segmentation branch. The first two branches belong to the faster R-CNN classification branch and the third branch is the fully-connected network (FCN) mask branch. Various sizes of feature patches are proposed after the above-mentioned procedures and resized to consistent sizes using another ROI Align operation, which can again retain more pixels than the ROI Pooling. For the faster R-CNN branch, the resized feature patches go through a convolution layer of 7 × 7 × 256 and two 1024-neuron fully-connected (FC) layers to predict object scores and refine object locations. As for the FCN branch, the patches undergo several convolution layers of 14 × 14 × 256 and a de-convolution layer of 28 × 28 × 256. Eighty 28 × 28 candidate masks are processed and rescaled according to the image size. Each pixel with the score being greater than 0.5 is assigned to the object of concern to generate the final binary mask, which is visualized together with the bounding box and class name.

### 2.5. General Workflow of Detector Training, Validation and Testing

**Figure 3:**
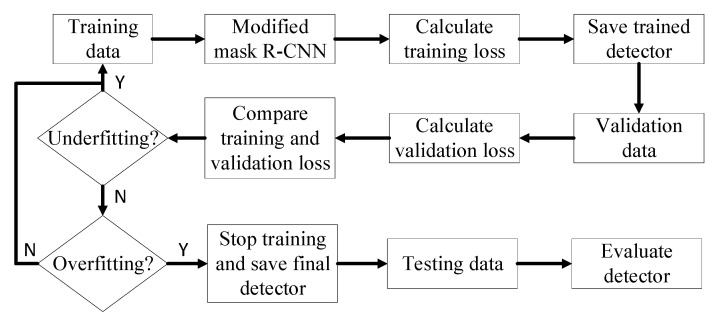
Illustration of the training, validation, and testing process. Mask R-CNN is mask region-based convolutional neural network. “Y” means that judgement is true and “N” means that judgement is false.

Figure 3 shows the overall process of training, validation and testing. Training data was input into the mask R-CNN detectors for training and the training loss was continuously calculated during the training process. The training detectors were stored in specific training iteration periodically and validated with the validation set. The training and validation losses were compared. If training and validation losses kept decreasing, it meant that the detectors were underfitted and needed more training. If the training loss decreased while the validation loss increased, it meant that the detectors were overfitted and the training process needed to be stopped [28]. With the final saved detectors, the hold-out testing data was used to evaluate the detector performance on preening detection. The computing system used for detector training, validation and testing computing was equipped with 32 GB RAM, Intel(R) Core (TM) i7-8700K processor and NVIDIA GeForce GTX 1080 GPU card (Dell Inc., Round Rock, TX, USA).

Table 1 shows the data distribution for training, validation and testing. Labeled images were described in Section 2.3 with 1175 images for training, 102 for validation and 423 for testing, resulting in 8464 labeled hens for training, 762 for validation and 2788 for testing. The training, validation and testing data came from three different days, respectively and those images had at least 1-min intervals. Therefore, they were thought to have sufficient variations for detector development.

**Figure 4:**
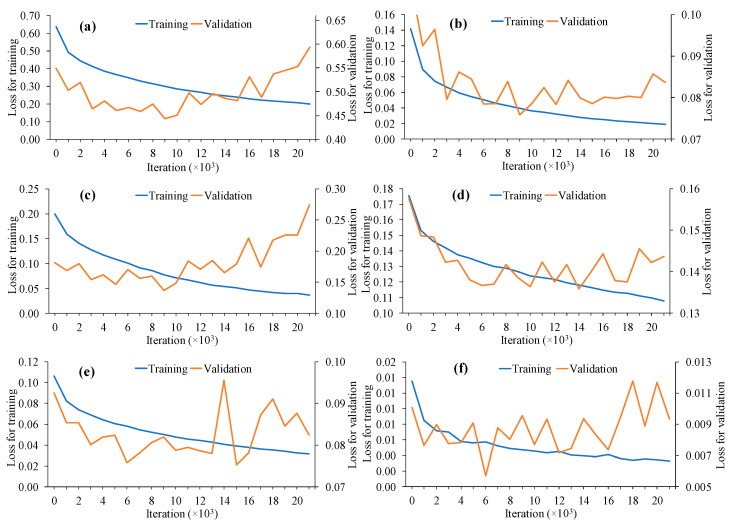
Samples of training and validation losses during training process. (**a**) Total loss; (**b**) detection head bounding box loss; (**c**) detection head class loss; (**d**) detection head mask loss; (**e**) region proposal network bounding box loss; and (**f**) region proposal network class loss.

The losses for training and validation included total loss, detection head class loss, detection head bounding box loss, detection mask loss, RPN bounding box loss and RPN class loss. The six types of losses were reported by He et al. [15] and reflected how much deviation there was between prediction and ground truth (Figure 4). Except for total loss, the other five types of losses corresponded to the three outputs in the detection head and two outputs in the RPN and the total loss was the sum of the five losses. A smaller loss indicated a better prediction. For instance, as loss samples shown in Figure 4, the training losses kept decreasing, while most of the validation losses decreased before 9 × 10^3^ iterations and had a rebound increase after 9 × 10^3^ iterations. Therefore, the training process was stopped at the 9 × 10^3^th iteration to avoid overfitting and the detectors were saved accordingly.

### 2.6. Modifications for Detector Development

The modifications for the detector development involved ResNet architecture, pre-trained weight, image resizer and number of ROI. The detectors with the following modifications was trained as described in Section 2.5 and the modification with optimal testing performance was used to develop the preening behavior detectors. The following modifications were trained with the default settings of mask R-CNN that were ResNet101, pre-trained COCO weights, ‘Square’ image resizer mode and 200 ROIs, unless specified in the sections. As for other hyperparameters for training, we followed the default settings recommended by Abdulla [22].

#### 2.6.1. Residual Network Architecture

**Figure 5:**
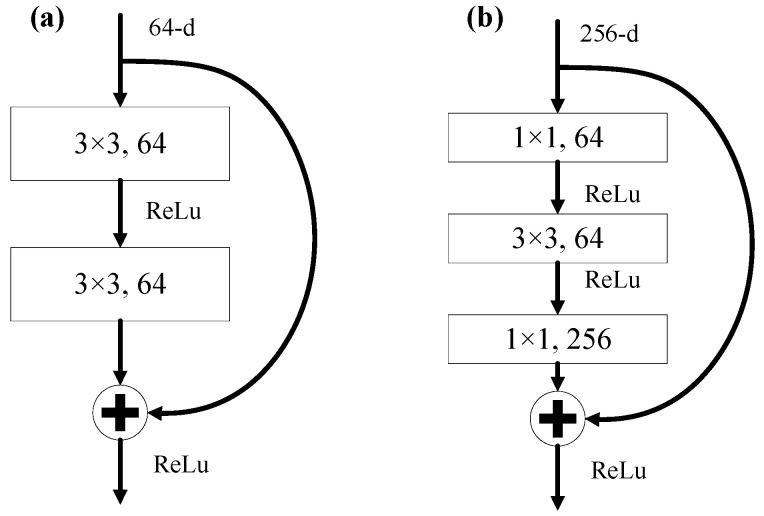
Examples of building block with shortcut connections for the residual network (ResNet). (**a**) Normal block at the C2 stage of the ResNet18 and ResNet34; and (**b**) bottleneck block at the C2 stage of the ResNet50-ResNet1000. ReLu is rectified linear units; 64-d is depth of 64; and 256-d is depth of 256. The figure was redrawn from He, et al. [29].

The ResNet was proposed by He, et al. [29]. Sufficiently extracting semantics in the C2–C5 stages was critical for detection performance. Six ResNet architectures that were ResNet18, ResNet34, ResNet50, ResNet101, ResNet152 and ResNet1000 were embedded into the mask R-CNN backbone for training (Table 2). The number beside ‘ResNet’ indicates the number of layers in the architecture. The ResNets with less than 50 layers were constructed with normal blocks (Figure 5a), while those with more than 50 layers were stacked with bottleneck blocks (Figure 5b), which can reduce computational complexity with increasing layers in the ResNet. The original mask R-CNN was built with ResNet50 or ResNet101.

#### 2.6.2. Pre-Trained Weight

The mask R-CNN was pre-trained with the benchmark datasets of COCO [18] and ImageNet [19] and obtained pre-trained weights. The trainings included without pre-trained weights, with pre-trained COCO weights and with pre-trained ImageNet weights. The training with the pre-trained weights only involved the heads of FPN, RPN and detection branches, which contained 28 items, while the full layer training without pre-trained weights was related to every layer in the detectors, which contained 236 items in total.

#### 2.6.3. Image Resizer

**Figure 6:**
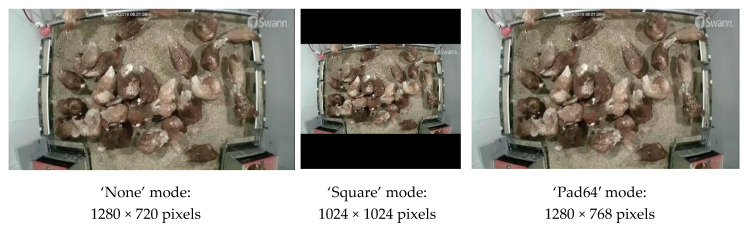
Resized sample images with three modes of image resizers.

To obtain uniform size of images for detector development, we need to resize the input images to the same size. Appropriate image resizers could improve processing speed and retain as much pixel-wise information as possible [22]. Three modes of resizers were compared, which were ‘None,’ ‘Square’ and ‘Pad64′. In the ‘None’ mode, input images (1280 × 720 pixels) were neither resized nor padded. In the ‘Square’ mode, input images were resized from 1280 × 720 pixels to 1024 × 1024 pixels and zeros were used to pad blank areas of resized images. In the ‘Pad64′ mode, input images were resized from 1280 × 720 pixels to 1280 × 768 pixels and the differences were padded with zeros. Resized sample images with the three modes of resizing are shown in Figure 6.

#### 2.6.4. Proposed Regions of Interest

Target preening birds may be ruled out in a feature map with insufficient ROIs, resulting in miss-identification of preening birds, while processing speed may decrease using a map with excessive ROIs [23]. The detectors were trained with 30, 100, 200, 300, 400 and 500 ROIs and the performance was compared.

### 2.7. Evaluation Metrics

After the detectors were trained and validated, the hold-out testing set was used for evaluating the trained detectors as described in Section 2.5. To determine whether a preening hen had been correctly segmented, the intersection over union (*IOU*) for each predicted hen was computed using overlap and union pixels of the ground truth and prediction (Equation (1)). An *IOU* greater than 0.5 in this case means the detectors segmented and detected a preening hen correctly.

(1) $$IOU\left[\%\right]=\;\frac{\left(pixels\;\in ground\;truth\right)\cap \left(pixels\;\in prediction\right)}{\left(pixels\;\in ground\;truth\right)\cup \left(pixels\;\in prediction\right)}\times 100\%$$

The mean *IOU* (MIOU) was used to evaluate overall segmentation performance of the detectors and calculated in Equation (2).

(2) $$MIOU=\;\frac{\sum_{i=1}^{n}IOU_{i}}{n}$$

where $IOU_{i}\;$is the *IOU* for the *i*th preening hen and *n* is the total number of preening hens.

Precision, recall, specificity, accuracy and F1 score for detecting each preening hen in the images were calculated using Equations (3)–(7). Precision is the percentage of true preening cases in all detected preening cases. Recall is the percentage of the true preening cases in all manually-labeled preening cases. Specificity is the percentage of true non-preening cases in all manually-labeled non-preening cases. Accuracy is the percentage of true preening and non-preening cases in all cases. F1 score is the harmonic mean of precision and recall and a balance metric on comprehensively evaluating false preening and non-preening cases. For all five metrics, a closer to 100% value reflects a better performance of the detectors.

(3) $$Precision\left[\%\right]=\;\frac{TP}{TP+FP}\times 100\%$$

(4) $$Recall\left[\%\right]=\;\frac{TP}{TP+FN}\times 100\%$$

(5) $$Specificity\left[\%\right]=\;\frac{TN}{TN+FP}\times 100\%$$

(6) $$Accuracy\left[\%\right]=\;\frac{TN+TP}{TN+TP+FN+FP}\times 100\%$$

(7) $$F1\;\mathrm{score}\;[\%]\;=2\times \frac{Precision\times Recall}{Precision+Recall}\times 100\%$$

where *TP* is true positive, that is, number of cases that a detector successfully detects existent preening hens in an image with *IOU* greater than 0.5; *FP* is false positive, that is, number of cases that a detector reports non-existent preening hens in an image or *IOU* is less than 0.5; *FN* is false negative, that is, number of cases that a detector fails to detect existent preening hens in an image; and *TN* is true negative, that is, number of cases that non-preening hens are reported by both a detector and manual label.

Average precision (*AP*) summarizes the shape of the precision-recall curve and is defined as the mean precision at a set of 11 equally-spaced recall levels [0, 0.1, …, 1] [30]. The precision-recall curve is produced according to the predicted confidence level. Increasing the confidence may reduce false positives but increase false negative, resulting in increasing precision and decreasing recall. A closer to 100% *AP* indicates a more generalized detector to detect objects with various confidence. The calculation of the *AP* is shown in Equation (8).

(8) $$AP\left[\%\right]=\frac{1}{11}\underset{r\in \left\{0,0.1,\ldots ,1\right\}}{\sum }P_{interp}\left(r\right)$$

where r is level of recall at {0,0.1,…,1}; and $P_{interp}\left(r\right)$ is the interpolated precision in the precision-recall curve when recall is r.

The interpolated precision is the maximum value within one piece of a wiggle-shape curve (Equation (9)).

(9) $$P_{interp}\left(r\right)=\underset{\tilde{r}:\tilde{r}\geq r}{\max}P\left(\tilde{r}\right)$$

where $\tilde{r}\;$is the recall within a wiggle piece; and $P\left(\tilde{r}\right)$ is the measured precision at recall $\tilde{r}$.

The processing time reported by Python 3.6 was used to evaluate the processing speed of the detectors for processing 423 images. The processing speed (ms·image^−1^) was obtained by dividing the total processing time with 423 images.

### 2.8. Sample Detection

We finally deployed the detector trained with ResNet101, pre-trained COCO weights, ‘Square’ mode and 200 ROIs, after the performance comparison. We continuously detected hen preening behaviors for half hour in week 38 of bird age. A segmented image based on traditional Otsu’s thresholding [31] was used to compare the result of preening instance segmentation using the mask R-CNN detector. The hen preening behaviors at 6:00 am–6:30 am were characterized as time spent preening (min∙hen^−1^), number of preening bouts (bouts∙hen^−1^), average preening duration (min∙bout^−1^), frequency of preening duration, number of birds simultaneously preening and spatial distribution of preening birds. Spatial location of preening birds was plotted in a heat map. To construct a heat map, a mesh grid was firstly constructed onto the pen map based on the dimension of the pen, in which the gird size was set to 10 pixels. Then a Standard Gaussian Kernel Density Estimation Function was run onto the center of each grid in the map and the preening frequency in each grid was calculated by Equation (10). Finally, the density map was visualized using Matplotlib, an open-source visualization library. The cooler-color (i.e., dark blue) areas in the map represented the areas where birds performed preening more often, while the warmer-color (i.e., dark red) areas were the areas where birds were less likely to preen.

(10) $$P=\overset{n}{\underset{i=1}{\sum }}\frac{1}{\sqrt{2\pi }}e^{-d_{i}^{2}/2}$$

where *P* is the probability in Standard Gaussian Distribution curve; *n* is the total number of grids in the entire image; and $d_{i}$ is the pixel-representing distance between the grid center and ith detected preening bird center.

## 3. Results

### 3.1. Sample Detection

**Figure 7:**
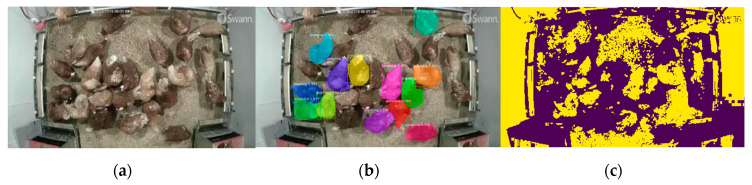
Sample detection. (**a**) Original image; (**b**) preening instance segmentation using the mask region-based convolution neural network detector; and (**c**) image segmentation using Otsu’s thresholding. Some preening birds in the Figure 7b are masked with different colors.

A sample detection is shown in Figure 7. Individual preening birds could be detected and segmented separately using the mask R-CNN detector, while some segmented hens by traditional thresholding method were cohesive and mixed with the background due to similar features with the background. Therefore, we applied the CNN detector to detect preening hens in this case. Meanwhile, as the deep learning outperformed the traditional image processing with regards to object segmentation, it may be a better choice for some research/application purposes (e.g., bird activity analysis).

### 3.2. Performance of Various Residual Networks

Table 3 shows the performance of various ResNets on preening detection. The six ResNets had similar segmentation performance of preening birds as indicated by similar MIOU (87.3–87.8%). The ResNet18 had middle performance but the second fastest processing speed (364.8 ms·image^−1^). The ResNet34 had the highest precision (88.5%) but the second lowest recall (86.2%) and AP (83.1%). The ResNet50 had the lowest recall (85.3%), accuracy (93.5%), F1 score (84.9%), AP (81.4%) and processing speed (342.9 ms·image^−1^). The ResNet101 had the highest accuracy (95.0%) and F1 score (88.1%). The ResNet152 had the lowest precision (83.1%) but the highest AP (85.7%). The ResNet1000 had the third lowest precision (84.5%) and slowest processing speed (393.2 ms·image^−1^). Overall, the six ResNets had strengths and weaknesses in different detection performance. Since the ResNet101 is a popular network in other agriculture applications, it was selected to develop the detector.

### 3.3. Performance of the Detectors Trained with Various Pre-Trained Weights

Table 4 shows the detection performance of the mask R-CNN detectors trained with various pre-trained weights. The detector trained with pre-trained ImageNet weights had the low performance of preening detection and segmentation among the three trainings, except for the specificity and precision. The detectors trained without pre-trained weights and with pre-trained COCO weights had similar detection performance, while the former training took ~50% more time and ~70% more computer memory. Therefore, the detector was trained with the pre-trained COCO weights in this case.

### 3.4. Performance of Various Image Resizers

Table 5 shows the performance of various image resizers. Similar MIOU and accuracy were observed for the three modes of resizers. The ‘Square’ mode had the lowest recall (86.3%) and F1 score (86.6%) but the highest specificity (96.6%). The ‘Pad64′ had the lowest precision (84.2%) and specificity (95.6%) but the highest recall (90.1%) and processing speed (383.3 ms·image^−1^). It should be noted that except for processing speed and recall, performance differences among the resizers were mostly less than 3%. As there was no obvious strength of detection performance among the resizers, the default resizers (‘Square’ mode) was deployed to develop the final detector.

### 3.5. Performance of the Detectors Trained with Various Numbers of Regions of Interest

The MIOU, accuracy and F1 score were similar among different numbers of ROIs (Table 6). The detector trained with 30 ROIs had the lowest recall (79.3%) and AP (75.8%) but the highest precision (92.5%) and specificity (98.2%). With more than 30 ROIs, the precision, recall, specificity and AP, respectively, ranged from 82.8–85.8%, 87.2–90.7%, 95.0–96.3% and 84.9–86.5%. The processing speed (378.2–390.7 ms·image^−1^) did not absolutely increase as more ROIs were used. Because there was no absolute improvement of detection performance with increasing ROIs (>30), the default ROIs of 200 was used in the final training.

### 3.6. Preening Behavior Measurement via the Trained Detector

**Figure 8:**
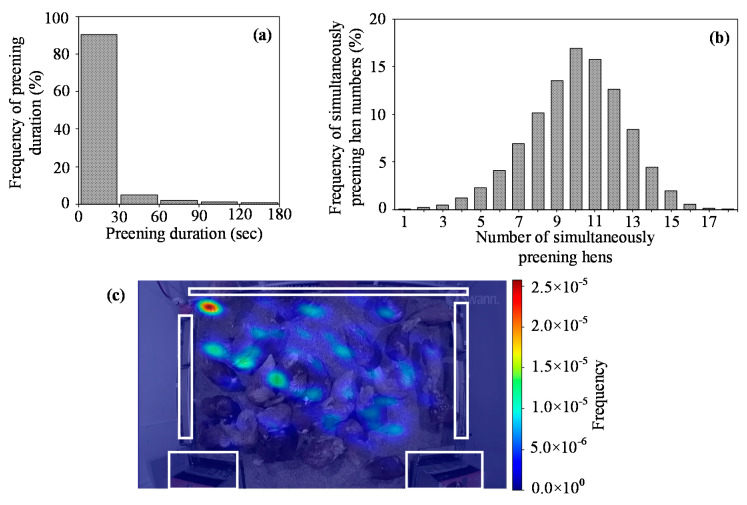
Preening behavior measurement during 6:00–6:30 am for 38-week-old hens. (**a**) Frequency of preening duration. (**b**) Frequency of simultaneously preening numbers. (**c**) Heat map for the location of preening birds. The drinker, feeder and nest box are marked as the white rectangles on the top, middle and bottom, respectively. The non-unit frequency represents the probability for birds preening at specific locations and is calculated by Standard Gaussian Kernel Density Estimation Function.

Figure 8 shows the preening behavior measurement in half hour via the trained detector. The hen spent on average 18.1 min, 106 bouts and 0.23 min∙bout^−1^ on preening. For over 90% of the time, the hens preened for less than 30 sec. A hen preened for up to 20.5 min within a preening event. Ten birds choosing to simultaneously preen took up the most proportion (16.9%) and the overall frequency was distributed in a shape of normal distribution. The hens spent more time preening at the top left corner of the pen. The hotspot was caused by multiple preening birds. Some birds may finish the preening and leave for eating/drinking while others may enter that area for preening.

## 4. Discussion

### 4.1. Ambiguous Preening Behavior

**Figure 9:**
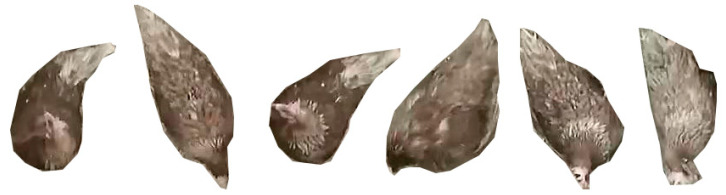
Sample pictures of questionable birds. The birds were manually cropped from original images. They were not labeled as preening birds and used for model development.

Hens can preen various parts of their body. In the images from the single camera and camera angle, some birds were hard to manually tell whether they were preening (Figure 9), which were ~3% of all hens in images. Those questionable hens were not labeled in this case and only the hens with clear preening features as mentioned in Figure 1 were labeled. Although this could make the detectors accurately detect preening hens with obvious features, the detectors still inevitably became ambiguous on detecting those questionable hens, especially the birds preening their chest or pecking ground. That could compromise detection performance. To reduce the confusion and improve detection performance, multiple cameras with multiple angles may be considered to capture different views of preening birds. To exemplify the applications of mask R-CNN on poultry preening behavior detection, we trained the detectors with images only containing brown hens. However, the detector may also be trained with other images containing other types of chickens, which can extend application range. Based on our previous experiment, deep learning networks could be generalized to different light intensities, backgrounds, object colors, object numbers and object sizes, as long as they were fed and trained with enough sample images [14].

As shown in Figure 8a, birds typically preened for less than 30 s and some birds even did it shorter (<1 s). With that regard, if the accuracy is acceptable, higher processing speed is still preferred since it may cover more prompt preening behaviors.

### 4.2. Segmentation Method Comparison

The compared segmentation method, despite not being the most state-of-the-art, is still commonly used for bird activity evaluation [11,32]. Pairs of adjacent images were compared to get the difference images and then the resulting images were binarized using the image processing methods. Activity index may reflect status of birds responding to surroundings and is important for poultry production. This parameter was extracted with traditional image segmentation methods. However, based on our current test, the traditional methods may result in cohesive maps. Although delicate adjustment may help to solve the issue, it requires intensive labor and has poor generalization. The mask R-CNN could solved the problem and may facilitate the bird activity evaluation, thus being recommended in this case.

### 4.3. Architecture Selection

The mask R-CNN is not the most state-of-the-art architecture. However, as agricultural engineers, our major goal is not to pursue the most advanced technique regardless but to seek reliable solutions to facilitate agricultural productions. With that regard, mask R-CNN was widely used in different areas and commercialized to accurately detect objects of concern, which may be acceptable for farmers, thus being our solution to detect poultry preening. Meanwhile, based on our preliminary test, the mask R-CNN outperformed its counterparts (e.g., SSD, faster R-CNN and R-FCN) in terms of accuracy, because the mask R-CNN retained as many pixels as possible using FPN and ROI align. Taking these into consideration, we decided to test the mask R-CNN systematically and seek the optimal modifications of the mask R-CNN.

### 4.4. Performance of Various Residual Network

Various ResNet architectures had different performance on preening detection. The efficiency of instance segmentation mainly relied on the mask detection architecture (FCN in this case) in the mask R-CNN detector [15,33] and the same mask detection architecture among various ResNets caused similar MIOU. Although the ResNet101 had slightly better or similar precision, recall, specificity, accuracy, F1 score and AP, the differences of the performance were small (2–4%). The ResNet-FPN backbone was proposed to extract ROI features from different levels according to their scales within an images and ResNet with more layers may theoretically improve the extraction and further detection performance [29,34]. The scales in some common images were partitioned at three levels that were <1024 pixels, 1024–9216 pixels and >9216 pixels corresponding to small, medium and large objects, respectively [35]. Compared with those, the scales of preening birds ranged from 7765 to 17,353 pixels, in which object areas in an image were relatively consistent. That may be the reason for why we cannot get significant improvement with ResNets having more layers.

Appropriate design of the CNN architecture could improve processing speed as more layers are stacked onto the architecture. The processing speed increased as the ResNet layers increased from 18 to 34 and 50 to 1000. However, it decreased as the ResNet increased from 34 layers to 50 layers. As shown in Figure 4, the ResNet18–ResNet34 were built with normal blocks, while the ResNet50–ResNet1000 were constructed with bottleneck blocks. The latter design can reduce the computational complexity and further increase the processing speed in deeper ResNets (≥50 layers) [29]. In sum, proper CNN architectures are critical for developing a robust and efficient detector as it can affect detection performance.

### 4.5. Performance of the Detectors Trained with Various Pre-Trained Weights

Modern CNNs are massive architectures containing considerable parameters to be trained, thus, efficiently training the inner structure is critical. Transfer learning could be a solution. Compared with the performance between the trainings without pre-trained weights and with pre-trained COCO weights, transfer learning could save training time without compromising detection performance. The latter training only involved the heads of FPN, RPN and detection branches, containing high-level semantics [36]. Such semantics may be more important for instance segmentation and object detection than low-level generic features extracted by the bottom architecture of the detectors [15]. That is why the detectors with the two trainings showed similar performance. As for transfer learning, various pre-trained weights among benchmark datasets can result in various performance. Perhaps, the pre-trained COCO weights had more similarity for preening hens than the pre-trained ImageNet weights, resulting in better efficiency of transfer learning and better performance for the former pre-trained weights [18,19,20].

### 4.6. Performance of Various Image Resizers

The resizers in this case had similar detection performance. The original image size was 1280 × 720 pixels and the size after resizing was 1024 × 1024 pixels for the ‘Square’ mode and 1280 × 768 pixels for the ‘Pad64′ mode. Most of the sizes were the multipliers of 64, which can ensure smooth scaling of feature maps up and down at the six levels of the FPN and reduce information loss [22]. That could result in the similar performance. Furthermore, the shapes of preening birds were not distorted before and after resizing, which made the detectors learn consistent features of preening birds and generate similar results. Reducing input image sizes indeed can help to cut processing time [23].

### 4.7. Performance of the Detectors Trained with Various Numbers of Regions of Interest

Detection performance varied with the proposed numbers of ROI. When less ROIs (<30) were proposed, some candidate preening hens may be ruled out, resulting in low recall and AP [23]. Meanwhile, fewer non-preening birds may be wrongly recognized as preening birds with less ROIs, causing higher precision and specificity. However, these trends disappeared when the ROIs were more than 100. Perhaps, more than 100 ROIs were sufficient to cover possible preening hens for the detection in this case. Processing speed did not absolutely increase with advanced ROIs, probably because processing lower than 500 ROIs did not exceed the capacity of the mask R-CNN detectors [15,23].

### 4.8. Preening Behavior Measurement with the Trained Detector

Individual preening hens could be continuously monitored with the trained detector. The extracted behavior information showed that the hens showed temporal and spatial preference on the preening during the testing period. These behaviors may provide valuable insights into farm management and facility design. For example, hens may show displacement preening around feeders when they cannot access feed [25] and understanding the frequency of preening hens present around feeders may help to evaluate the sufficiency of feeder allowance. At the current stage, we just explored the probability of using deep learning to detect the preening behavioral responses and further research is recommended to determine the thresholds of the responses with regards to welfare evaluation. Overall, the mask R-CNN preening behavior detector is a useful tool to evaluate hen preening behaviors.

## 5. Conclusions

This study developed mask R-CNN preening behavior detectors by modifying the ResNets, pre-trained weights, image resizers and number of ROIs. The detectors accurately segmented individual preening hens (MIOU: 83.6–88.7%) and had decent performance on detecting preening and non-preening hens, in which the precision, recall, specificity, accuracy, F1 score and AP were mostly over 80%. The overall processing speed for preening detection ranged from 342.9 to 413.7 ms·image^−1^. The ResNet101 performed better on the preening detection among the six ResNets. The pre-trained COCO weights had better transfer learning efficiency than the pre-trained ImageNet weights. The image resizers in the ‘None,’ ‘Square’ and ‘Pad64′ modes performed similarly on hen preening detection. The 30 ROIs had the highest precision and specificity but the lower recall and AP among various numbers of ROIs, while more than 100 ROIs had similar performance on hen preening detection. With the trained detector, temporospatial preening behaviors of individual hens could be extracted. Overall, the mask R-CNN preening behavior detector is a useful tool to detect hen preening behaviors.

## Figures and Tables

**Table 1:** Data distribution for training, validation and testing.

Items	Training	Validation	Testing
Hen age (day)	266	267	268
Images	1175	102	423
Number of preening hens	8464	762	2788
Number of non-preening hens	26,786	2298	9902

**Table 2:** Detailed architectures for residual network (ResNet).

Stage of Convolution	ResNet18	ResNet34	ResNet50	ResNet101	ResNet152	ResNet1000
C1	7 × 7, 64, stride 2 3 × 3 max pooling, stride 2					
C2	$\left[\begin{array}{c} 3\times 3,\;64\\ 3\times 3,\;64 \end{array}\right]\times 2$	$\left[\begin{array}{c} 3\times 3,\;64\\ 3\times 3,\;64 \end{array}\right]\times 3$	$\left[\begin{array}{c} 1\times 1,\;64\\ 3\times 3,\;64\\ 1\times 1,\;256 \end{array}\right]\times 3$	$\left[\begin{array}{c} 1\times 1,\;64\\ 3\times 3,\;64\\ 1\times 1,\;256 \end{array}\right]\times 3$	$\left[\begin{array}{c} 1\times 1,\;64\\ 3\times 3,\;64\\ 1\times 1,\;256 \end{array}\right]\times 3$	$\left[\begin{array}{c} 1\times 1,\;64\\ 3\times 3,\;64\\ 1\times 1,\;256 \end{array}\right]\times 248$
C3	$\left[\begin{array}{c} 3\times 3,\;128\\ 3\times 3,\;128 \end{array}\right]\times 2$	$\left[\begin{array}{c} 3\times 3,\;128\\ 3\times 3,\;128 \end{array}\right]\times 4$	$\left[\begin{array}{c} 1\times 1,\;128\\ 3\times 3,\;128\\ 1\times 1,\;512 \end{array}\right]\times 4$	$\left[\begin{array}{c} 1\times 1,\;128\\ 3\times 3,\;128\\ 1\times 1,\;512 \end{array}\right]\times 4$	$\left[\begin{array}{c} 1\times 1,\;128\\ 3\times 3,\;128\\ 1\times 1,\;512 \end{array}\right]\times 22$	$\left[\begin{array}{c} 1\times 1,\;128\\ 3\times 3,\;128\\ 1\times 1,\;512 \end{array}\right]\times 248$
C4	$\left[\begin{array}{c} 3\times 3,\;256\\ 3\times 3,\;256 \end{array}\right]\times 2$	$\left[\begin{array}{c} 3\times 3,\;256\\ 3\times 3,\;256 \end{array}\right]\times 6$	$\left[\begin{array}{c} 1\times 1,\;256\\ 3\times 3,\;256\\ 1\times 1,\;1024 \end{array}\right]\times 6$	$\left[\begin{array}{c} 1\times 1,\;256\\ 3\times 3,\;256\\ 1\times 1,\;1024 \end{array}\right]\times 23$	$\left[\begin{array}{c} 1\times 1,\;256\\ 3\times 3,\;256\\ 1\times 1,\;1024 \end{array}\right]\times 22$	$\left[\begin{array}{c} 1\times 1,\;256\\ 3\times 3,\;256\\ 1\times 1,\;1024 \end{array}\right]\times 247$
C5	$\left[\begin{array}{c} 3\times 3,\;512\\ 3\times 3,\;512 \end{array}\right]\times 2$	$\left[\begin{array}{c} 3\times 3,\;512\\ 3\times 3,\;512 \end{array}\right]\times 3$	$\left[\begin{array}{c} 1\times 1,\;512\\ 3\times 3,\;512\\ 1\times 1,\;2048 \end{array}\right]\times 3$			$\left[\begin{array}{c} 1\times 1,\;512\\ 3\times 3,\;512\\ 1\times 1,\;2048 \end{array}\right]\times 247$
	Average pooling, 1000-d FC, softmax					

**Note:** ResNet18-ResNet1000 are residual network with 18–1000 layers of convolution; C1–C5 are convolutional stages 1 to 5 in the ResNet; and FC is fully-connected.

**Table 3:** Performance of various residual networks on preening detection.

ResNet	MIOU (%)	Precision (%)	Recall (%)	Specificity (%)	Accuracy (%)	F1 Score (%)	AP (%)	Processing Speed (ms·image^−1^)
ResNet18	87.4	87.2	87.1	96.7	94.7	87.1	83.6	364.8
ResNet34	87.4	88.5	86.2	97.0	94.8	87.3	83.1	378.4
ResNet50	87.8	84.4	85.3	95.7	93.5	84.9	81.4	342.9
ResNet101	87.4	87.7	88.4	96.7	95.0	88.1	83.5	386.0
ResNet152	87.4	83.1	90.1	95.1	94.1	86.5	85.7	387.7
ResNet1000	87.4	84.5	90.8	95.6	94.6	87.6	85.6	393.2

**Note:** ResNet is residual network; ResNet18-ResNet1000 is the ResNet with 18–1000 layers; MIOU is mean intersection over union; and AP is average precision.

**Table 4:** Performance of the detectors trained with various pre-trained weights.

Training	MIOU (%)	Precision (%)	Recall (%)	Specificity (%)	Accuracy (%)	F1 Score (%)	AP (%)	Processing Speed (ms·image^−1^)
w/o pre-trained weights	88.7	80.3	92.3	93.9	93.6	85.9	87.5	379.0
w/pre-trained COCO weights	87.2	83.4	91.3	94.5	93.8	87.2	86.7	382.9
w/pre-trained ImageNet weights	83.6	81.2	83.1	94.9	92.5	82.2	80.0	413.7

**Note:** COCO is common object in context; MIOU is mean intersection over union; and AP is average precision. ‘w/o’ and ‘w/’ indicate ‘without’ and ‘with’, respectively.

**Table 5:** Performance of various image resizers.

Mode of Image Resizer	MIOU (%)	Precision (%)	Recall (%)	Specificity (%)	Accuracy (%)	F1 Score (%)	AP (%)	Processing Speed (ms·image^−1^)
None	87.2	85.3	88.4	96.0	94.4	86.8	84.6	377.7
Square	87.6	86.9	86.3	96.6	94.5	86.6	86.7	377.8
Pad64	87.0	84.2	90.1	95.6	94.5	87.0	86.3	383.3

**Note:** MIOU is mean intersection over union; and AP is average precision.

**Table 6:** Performance of the detectors trained with various numbers of regions of interest (ROI).

Number of ROI	MIOU (%)	Precision (%)	Recall (%)	Specificity (%)	Accuracy (%)	F1 Score (%)	AP (%)	Processing Speed (ms·image^−1^)
30	87.5	92.5	79.3	98.2	94.2	85.4	75.8	378.2
100	87.1	84.2	89.1	95.5	94.2	86.6	84.9	379.4
200	86.9	85.8	89.5	96.0	94.6	87.6	85.4	378.1
300	87.2	85.7	87.2	96.3	94.4	86.4	83.8	390.7
400	87.6	82.7	90.3	95.0	94.0	86.3	86.5	382.0
500	87.0	82.8	90.7	95.0	94.1	86.6	86.2	378.8

**Note:** MIOU is mean intersection over union; and AP is average precision.

## Nomenclature

AP	Average precision	MIOU	Mean intersection over union
ARS	Agriculture Research Service	MP	Max pooling
Bbox	Bounding box	NMS	Non-maximum suppression
Box1–Box5	Proposed boxes with various sales and ratios after the region proposal network	$P_{interp}\left(r\right)$	Interpolated precision in the precision-recall curve when recall is *r*
C1–C5	Convolutional stages 1 to 5 in the residual network	$P\left(\tilde{r}\right)$	Measured precision at recall $\tilde{r}$
COCO	Common object in context	P2-P6	Feature maps in the feature pyramid network
Conv.	Convolution	ReLu	Rectified linear units
CNN	Convolutional neural network	ResNet	Residual network
Faster RCNN	Faster region-based convolutional neural network	ResNet18-ResNet1000	Residual network with 18-1000 layers of convolution
FC	Fully-connected	ROI	Regions of interest
FCN	Fully-connected network	RPN	Region proposal network
FN	False negative	TN	True negative
FP	False positive	TP	True positive
FPN	Feature pyramid network	Ups.	Upsampling
IOU	Intersection over union	USA	United State of American
Mask R-CNN	Mask region-based convolutional neural network	USDA	United State department of agriculture
